# Development of Antibody-Fragment–Producing Rice for Neutralization of Human Norovirus

**DOI:** 10.3389/fpls.2021.639953

**Published:** 2021-02-23

**Authors:** Ai Sasou, Yoshikazu Yuki, Shiho Kurokawa, Shintaro Sato, Yuki Goda, Masao Uchida, Naomi Matsumoto, Hiroshi Sagara, Yuji Watanabe, Masaharu Kuroda, Naomi Sakon, Kotomi Sugiura, Rika Nakahashi-Ouchida, Hiroshi Ushijima, Kohtaro Fujihashi, Hiroshi Kiyono

**Affiliations:** ^1^Division of Mucosal Immunology, IMSUT Distinguished Professor Unit, The Institute of Medical Science, The University of Tokyo, Tokyo, Japan; ^2^Division of Mucosal Vaccines, International Research and Development Center for Mucosal Vaccines, The Institute of Medical Science, The University of Tokyo, Tokyo, Japan; ^3^Mucosal Vaccine Project, BIKEN Innovative Vaccine Research Alliance Laboratories, Research Institute for Microbial Diseases, Osaka University, Osaka, Japan; ^4^Department of Immunology and Genomics, Graduate School of Medicine, Osaka City University, Osaka, Japan; ^5^Drug Discovery Research, Astellas Pharma Inc., Tsukuba, Japan; ^6^Medical Proteomics Laboratory, The Institute of Medical Science, University of Tokyo, Tokyo, Japan; ^7^The National Agriculture and Food Research Organization, Tsukuba, Japan; ^8^Department of Microbiology, Osaka Institute of Public Health, Osaka, Japan; ^9^Department of Pathology and Microbiology, Nihon University School of Medicine, Tokyo, Japan; ^10^Division of Clinical Vaccinology, International Research and Development Center for Mucosal Vaccines, The Institute of Medical Science, The University of Tokyo, Tokyo, Japan; ^11^Department of Pediatric Dentistry, The University of Alabama at Birmingham, Birmingham, AL, United States; ^12^Department of Immunology, Graduate School of Medicine, Chiba University, Chiba, Japan; ^13^Division of Gastroenterology, Department of Medicine, Chiba University – University of California San Diego Center for Mucosal Immunology, Allergy, and Vaccine, University of California, San Diego, San Diego, CA, United States

**Keywords:** norovirus, variable-domain llama heavy-chain antibody fragment, virus-like particle, transgenic rice, MucoRice

## Abstract

Human norovirus is the leading cause of acute nonbacterial gastroenteritis in people of all ages worldwide. Currently, no licensed norovirus vaccine, pharmaceutical drug, or therapy is available for the control of norovirus infection. Here, we used a rice transgenic system, MucoRice, to produce a variable domain of a llama heavy-chain antibody fragment (VHH) specific for human norovirus (MucoRice-VHH). VHH is a small heat- and acid-stable protein that resembles a monoclonal antibody. Consequently, VHHs have become attractive and useful antibodies (Abs) for oral immunotherapy against intestinal infectious diseases. MucoRice-VHH constructs were generated at high yields in rice seeds by using an overexpression system with RNA interference to suppress the production of the major rice endogenous storage proteins. The average production levels of monomeric VHH (7C6) to GII.4 norovirus and heterodimeric VHH (7C6-1E4) to GII.4 and GII.17 noroviruses in rice seed were 0.54 and 0.28% (w/w), respectively, as phosphate buffered saline (PBS)-soluble VHHs. By using a human norovirus propagation system in human induced pluripotent stem-cell-derived intestinal epithelial cells (IECs), we demonstrated the high neutralizing activity of MucoRice expressing monomeric VHH (7C6) against GII.4 norovirus and of heterodimeric VHH (7C6-1E4) against both GII.4 and GII.17 noroviruses. In addition, MucoRice-VHH (7C6-1E4) retained neutralizing activity even after heat treatment at 90°C for 20 min. These results build a fundamental platform for the continued development of MucoRice-VHH heterodimer as a candidate for oral immunotherapy and for prophylaxis against GII.4 and GII.17 noroviruses in not only healthy adults and children but also immunocompromised patients and the elderly.

## Introduction

Human norovirus infection is common in both developed and developing countries and is associated with severe complications in children younger than 5 years, elderly adults, and immunocompromised patients (Lopman et al., 2016). Although noroviruses are categorized into seven genogroups according to their capsid sequences, only viruses in the GI and GII genogroups, which comprise 28 genotypes, can infect humans (Vinjé, 2015). In the past decade, members of the GII.4 genotype have been major causative viruses, whereas GII.17 genotype viruses followed by GII.2 isolates have recently become the predominant strains in some parts of Asia, including Japan (Matsushima et al., 2015; Thongprachum et al., 2017). Currently, no licensed human norovirus vaccine, pharmaceutical drug, or therapy is available, although vaccine candidates against genotypes GI.1 and GII.4 are under development. Vaccination with the GII.4 virus-like particle (VLP) vaccine elicits antibodies (Abs) against several GII.4 strains, but the vaccine fails to induce any antibodies that block GII noroviruses other than those within the GII.4 genotype, including non-vaccine GII.4 strains (Kim et al., 2018; Leroux-Roels et al., 2018). Although the strategy for developing VLP-based vaccines needs further refinement, whether VLP vaccination is effective and safe for inducing antibodies against human norovirus in immunocompromised patients, infants, and the elderly remains unknown (Leroux-Roels et al., 2018).

The variable-domain llama heavy-chain antibody fragment, commonly called a “nanoantibody,” consists of a single-chain antibody that is produced by llamas and camels and that has a molecular weight of only 15,000 Da (Hamers-Casterman et al., 1993). VHHs are heat-stable and resistant to pepsin and acid (van der Linden et al., 1999). Because of these unique biological characteristics, VHHs are considered to be attractive and useful Abs for oral passive immunotherapy better than that achieved with oral administration of polyclonal Abs (Sarker et al., 1998, 2001). In addition, VHHs can be used safely and effectively in high-risk groups, such as hospitalized children, the elderly, and immunocompromised persons (Tremblay et al., 2013; Moonens et al., 2015). In fact, ARP1 – an orally administered rotavirus VHH made from yeast – was found to be safe and effective in reducing the severity of rotavirus-induced diarrhea in children in a phase II clinical trial conducted in Bangladesh (Sarker et al., 2013).

In addition to the yeast-based VHH production system, we previously developed transgenic rice that expresses VHH – the MucoRice-VHH system – as an oral immune-therapy platform (Tokuhara et al., 2013). In a previous study, we successfully expressed large amounts of ARP1 – a rotavirus-specific VHH – in rice seeds by using an overexpression system that included RNAi to suppress the production of major rice endogenous storage proteins such as prolamin and glutelin (MucoRice-ARP1; Tokuhara et al., 2013). Our study further demonstrated the heat and acid stability of MucoRice-ARP1. Similar to other MucoRice-expressed vaccine antigens such as the cholera toxin B subunit (Nochi et al., 2007; Yuki et al., 2013), MucoRice-ARP1 can be stored at room temperature for as long as 2 years, thus qualifying the product for refrigeration- or cold-chain–free production, storage, and delivery (Tokuhara et al., 2013).

By using phage-display technology, we recently obtained several VHH clones from llamas immunized with VLPs of either GII.4 or GII.17 norovirus and selected VHH clones 7C6 and 1E4, which specifically neutralized GII.4 and GII.17 noroviruses, respectively (Yuki et al., 2020). In the present study, we inserted the VHH clones 7C6 and 1E4 genes individually, and in combination, into the rice expression system and developed monomeric MucoRice-VHH 7C6 and heterodimeric MucoRice-VHH 7C6-1E4 for the control of norovirus infections. Because a suitable animal model for human norovirus infection is unavailable, we used human intestinal epithelial cells (IECs) derived from induced pluripotent stem cells (iPSCs; Sato et al., 2019) to assess whether the two forms of MucoRice-VHH could block the invasion of norovirus into human IECs. Our results showed that the MucoRice-VHH heterodimer (7C6-1E4) neutralized both GII.4 and GII.17 noroviruses, whereas the MucoRice-VHH monomer (7C6) neutralized GII.4 only. In addition, regarding neutralizing activity against GII.4 norovirus, the MucoRice-VHH 7C6-1E4 heterodimer was more effective than the MucoRice-VHH 7C6 monomer. Overall, our study has demonstrated promising evidence in support of continued advancement of MucoRice-VHH – especially the heterodimeric form of 7C6-1E4 – as a new generation of oral immunotherapeutic antibody drug for the control of norovirus infections.

## Materials and Methods

### Variable Domain of a Llama Heavy-Chain Antibody Fragments

Production of VHHs to GII.4_2006b (Sakai08-408) and GII.17 Kawasaki308 (OsakaFB16421) VLPs was outsourced (QVQ BV, Utrecht, The Netherlands; see Supplementary Table 1). We then generated a recombinant homodimeric VHH, which comprised two VHH 7C6 conjugated with a flexible (GGGGS)3 linker, and heterodimeric VHH, which comprised a single VHH 1E4 at the C-terminus and a VHH 7C6 at the N-terminus; the two VHHs were separated by a flexible (GGGGS)4 linker.

The DNA sequences of the homodimer and heterodimer were synthesized and inserted into the pET-20b (+) expression vector (Takara Bio). The resulting plasmids were transformed into Rosetta 2 (DE3) pLysS cells (Novagen, Madison, WI); the cells were cultured, harvested, and sonicated in sodium phosphate buffer containing 40 mM imidazole and protease inhibitor (cOmplete, Roche Diagnostics, Tokyo, Japan). The homodimer and heterodimer were purified from the supernatant by using an Ni Sepharose Fast Flow column (GE Healthcare, Chicago, IL) under solubilizing conditions (Supplementary Figure 1).

### DNA Constructs and Transformation of Rice Plants

**Figure 1:**
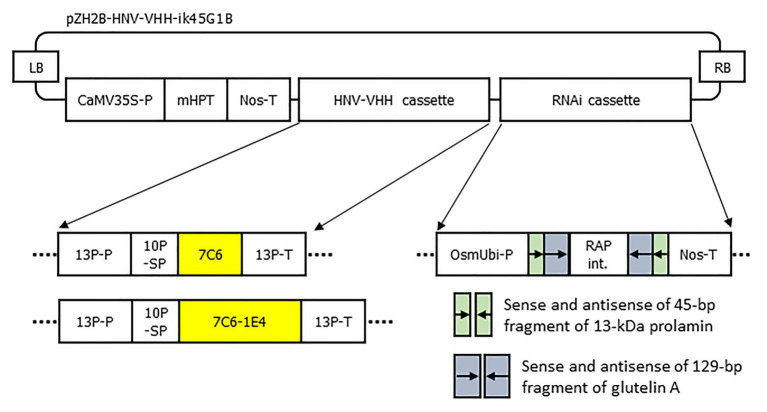
Schematic representation of the T-DNA region for the expression of 7C6 and 7C6-1E4 and the suppression of 13-kDa prolamin and glutelin A. LB, T-DNA left border; CaMV35S-P, cauliflower mosaic virus 35S promoter; mHPT, hygromycin phosphotransferase; Nos-T, nopaline synthase terminator; 13P-P, 13-kDa prolamin promoter; 10P-SP, 10-kDa prolamin signal peptide; 7C6, VHH against GII.4 norovirus; 7C6-1E4, heterodimer of 7C6 and 1E4 against GII.17 norovirus; 13P-T, 13-kDa prolamin terminator; OsmUbi-P, rice (*Oryza sativa*) modified polyubiquitin promoter; RAP int., rice aspartic protease intron; and RB, T-DNA right border.

The genes encoding 7C6 and 1E4 were selected as described previously (Yuki et al., 2020). The sequences of 7C6 and 7C6-(GGGGS)_3_-1E4(heterodimer) were synthesized with optimized codon usage for plants by Takara Bio (Shiga, Japan) and inserted into a binary T-DNA vector (pZH2Bik45G1B; Kuroda et al., 2010). This vector contains a cassette for the overexpression of VHH and a combination cassette for RNAi suppression of production of the major rice endogenous storage proteins, 13-kDa prolamin (RM1: Os07g0206500; coding sequence positions 1–45) and glutelin (GluA: Os10g0400200; coding sequence positions 142–270; Kuroda et al., 2010; Figure 1). We transformed a japonica rice plant cultivar, Nipponbare, with these plasmids by using an Agrobacterium-mediated method (Hiei et al., 1994). Plants that arose after greening from transgenic rice calli were transplanted into soil and maintained in a growth chamber (Panasonic, Kadoma, Osaka, Japan) on a 27°C, 12-h day: 22°C, 12-h night cycle. Several independent MucoRice-VHH lines were generated for 7C6 and 7C6-1E4 of MucoRice-VHH, and the VHH accumulation levels in seeds were determined by SDS-PAGE densitometric analysis or Western blotting or both. For each MucoRice-VHH, the plant line with the highest levels of VHH accumulated in the seed was selected and advanced to the T3 generation through self-crossing to obtain homozygous lines.

### Protein Analyses

Total protein was extracted from MucoRice-VHH powder by using a buffer containing 2% (wt/vol) SDS, 6% (wt/vol) β-mercaptoethanol, 50 mM Tris-HCl (pH 6.8), and 10% (wt/vol) glycerol. In addition, VHHs were extracted from MucoRice-VHH powder by using PBS with rotation at 4°C for 3 h. After centrifugation, the supernatants were analyzed by using SDS-PAGE and western blotting. Proteins were separated by using SDS-PAGE, and the gels were either stained with Coomassie Brilliant Blue (CBB) or transferred to polyvinylidene difluoride membrane (GE Healthcare). The VHH content in the PBS solution was determined through densitometry analysis of a CBB-stained SDS-PAGE gel; recombinant 7C6 or 7C6-1E4 heterodimer protein was used as a standard. The protein concentrations of the purified recombinant 7C6 and 7C6-1E4 from *Escherichia coli* were determined according to the theoretical absorbance at a wavelength of 280 nm, as determined from the respective amino-acid sequences (7C6: 1.774; 7C6-1E4: 1.813) and calculated by using the ProtParam tool.^1^

### Immunofluorescence Microscopy

Mature seeds were embedded by using a previously described method, with some modification (Nochi et al., 2007). Semi-thin (1 μm) sections of mature seeds were cut by using a diamond knife on an ultramicrotome (model EM UC6, Leica, Wetzlar, Germany). For double staining of prolamin and glutelin, the sections were blocked with 1% bovine serum albumin (BSA) in PBS for 1 h and incubated with rabbit anti-13 kDa-prolamin antibody (1:1,000) or mouse anti-glutelin antibody (5 μg/ml) for 1 h. The sections were washed with PBS and incubated with Cy3-conjugated anti-rabbit IgG antibody (1:200) or DyLight488-conjugated anti-mouse IgG antibody (1:200) in 1% BSA in PBS for 1 h; this was followed by washes with PBS.

For double staining of 7C6 and glutelin, the sections were blocked with 1% BSA in PBS for 60 min and incubated with rabbit anti-13 7C6 antibody (10 μg/ml) or mouse anti-glutelin antibody (5 μg/ml) for 1 h. The sections were washed with PBS and incubated with Cy3-conjugated anti-rabbit IgG antibody (1:200) or DyLight488-conjugated anti-mouse IgG antibody (1:200) in 1% BSA in PBS for 1 h; this was followed by washes with PBS. Images were captured by using a confocal laser scanning microscope (LSM 800 Axio Observer, Carl Zeiss, Oberkochen, Germany).

The fluorescence intensities of 13-kDa prolamin and glutelin were measured by using the mean gray value tool in ImageJ/Fiji free software.^2^ This tool sums the gray values of all pixels in the selected and then divides this value by the number of pixels. To calculate the relative value, each color (magenta or green) image was divided into four regions and converted to eight-bit gray images. The average of the mean gray values from the four component images was used to calculate the relative values of 13-kDa prolamin and glutelin when the average of the wild type (WT) mean gray value was defined as 1.

### Immunoelectron Microscopy

The distribution of VHH in rice seeds was analyzed by using immuno-transmission electron microscopy as previously described (Tokuhara et al., 2013). Ultrathin sections (150 nm) of immature seeds (14 days after flowering) were blocked with 10% goat serum in PBS and stained with rabbit anti-glutelin antibody, anti-prolamin antibody, and anti-7C6 antibody as described earlier for immunofluorescence microscopy. The reacted sections were incubated with gold particle-conjugated (18 nm) goat anti-mouse IgG and gold particle-conjugated (18 nm) goat anti-rabbit IgG antibody (Jackson ImmunoResearch Laboratories, West Grove, PA). Then, the reaction sections were stained with 2% (w/v) uranyl acetate and Reynolds’ lead citrate solution and observed under a transmission electron microscope (JEM-1400Flash, JEOL, Tokyo, Japan) at 80 kV.

### Neutralization Assay of Human Noroviruses

Variable domain of a llama heavy-chain antibody fragments were extracted from MucoRice-VHH powder into PBS (250 mg rice powder/ml) by rotating at 4°C for 3 h. After centrifugation, the supernatant (rice water) was passed through a 0.22-μm membrane. Neutralization assay of human noroviruses was performed as described previously (Yuki et al., 2020). Culture and passage of human iPSC-derived IECs in Matrigel (Corning, Corning, NY) and preparation of mono-layered IECs were performed as described previously (Sato et al., 2019). Each virus solution (Supplementary Table 2) was diluted to 1.5 × 10^6^ (GII.4_2006b) or 2.0 × 10^6^ (other genotypes) genome equivalents per 100 μl with and without VHH (5 μg or indicated amount) in base medium [advanced Dulbecco’s modified Eagle medium/F12 supplemented with 10-mM HEPES (pH 7.3), 2 mM Glutamax, and 100 units/ml penicillin plus 100-μg/ml streptomycin] and then incubated for 90 min. The prepared IECs (3–6 wells per sample) were inoculated with 100 μl of diluted virus solutions and left for 3 h in a 5% CO_2_ incubator at 37°C. The inoculum was then removed, and the cells were washed twice with 150 μl of base medium. We then added 100 μl of differentiation medium with 0.03% bile to the cells, gently pipetted them up and down twice, and collected them. This step was repeated, and the suspensions were pooled and collected as 3-h post-infection reference samples (total, 200 μl). Another 100 μl of differentiation medium [base medium composition: 1 × B-27 base medium, 1.25% fetal bovine serum (Biosera), 50 ng/ml mouse EGF, 375 ng/ml mouse R-Spondin 1 (R&D Systems), 50 ng/ml mouse Noggin (Peprotech), and 500 nM A83-01] with 0.03% bile was added to each well, and the mixtures were then cultured for 48 h in a 5% CO_2_ incubator at 37°C. The supernatants were collected, with one wash, in the same way as the reference samples (total, 200 μl). For the heat-treatment study, rabbit anti-GII.4 IgG, rabbit anti-GII.17 IgG, and heterodimeric VHHs (each 0.1 mg/ml in PBS) were heat-treated to 90°C for 10–20 mins by using a heat-block (Astec, Japan) and were then used in neutralization assays of human noroviruses, as described earlier. The inhibitory activity of heat-treated VHH or rabbit anti-norovirus IgG Ab against human norovirus replication is shown as the percentage of inhibitory activity in relation to that of untreated VHH or rabbit anti-norovirus IgG Ab.

### Statistical Analysis

Results were compared by using unpaired two-tailed Student’s *t*-tests. All statistical analyses were done by using Prism 7 (GraphPad Software, San Diego, CA).

## Results

### Expression of 7C6 and 7C6-1E4 Heterodimer in Transgenic Rice Seeds

**Figure 2:**
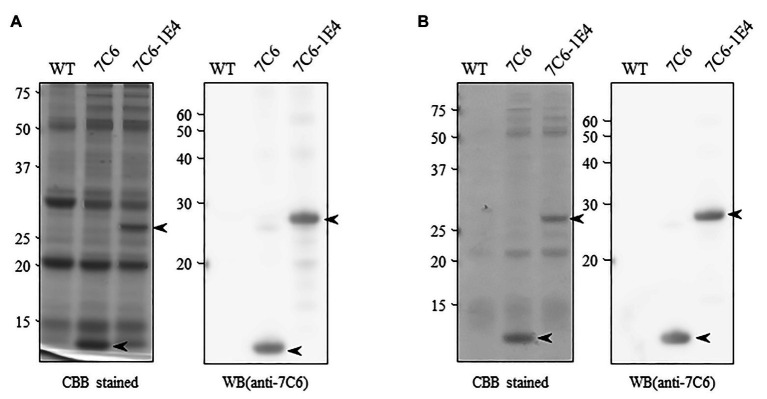
Expression of VHH in transgenic rice seeds. **(A)** Total protein extracted from seed flour of wild type (WT; Nipponbare), 7C6, and 7C6-1E4 rice. Arrowheads indicate the bands of VHHs. Left panel shows the Coomassie Brilliant Blue (CBB)-stained gel; right panel indicates the membrane immunoblotted by using anti-7C6 antibody. **(B)** Soluble protein extracted with phosphate buffered saline (PBS) from seed flour of WT, 7C6, and 7C6-1E4 rice. Arrowheads indicate the bands of VHHs. Left panel shows the CBB-stained gel; right panel indicates the membrane immunoblotted by using anti-7C6 antibody.

To develop MucoRice-VHH against norovirus, we inserted monomeric 7C6 VHH or the heterodimeric 7C6-1E4 VHH gene between the endosperm-specific 13-kDa prolamin promoter (13P-P) – combined with a 10-kDa prolamin signal peptide (10P-SP) gene – and the 13-kDa prolamin terminator (13P-T; Figure 1). In addition, we introduced a combination cassette for RNAi suppression of the 13-kDa prolamin and glutelin A rice storage proteins into T-DNA vector, to increase the expression level of VHH in rice seed (Figure 1). We transformed the plasmids into japonica rice plants (Nipponbare) by using Agrobacterium-mediated transformation and established the lines with the highest expression of each MucoRice-VHH. SDS-PAGE analysis showed that the monomeric 7C6 MucoRice-VHH generated a band with a molecular weight of 13.6 kDa on CBB-stained gels; the molecular weight of the heterodimeric MucoRice-VHH band was 27.3 kDa; these molecular weights were confirmed through western blotting with rabbit antibody against 7C6-VHH (Figure 2A).

By using densitometric analysis of SDS-PAGE gels, we determined that the expression levels of recombinant 7C6 VHH and 7C6-1E4 VHH reached averages of 7.17 and 4.63 mg/g seed, respectively. These VHHs were released easily from MucoRice-VHH powder by using PBS at room temperature (rice water; Figure 2B). The average yields of soluble 7C6 VHH and 7C6-1E4 heterodimeric VHH were 5.39 and 2.78 mg/g of rice water, corresponding to PBS extraction efficiencies of 75.1 and 59.9%, respectively (Table 1).

**Table 1:** Accumulation of MucoRice-variable domain of a llama heavy-chain antibody fragment (VHH).

	Concentration of VHH in PBS (mg/ml)	Proportion of VHH in PBS-soluble protein (%)	PBS-soluble VHH in rice seed (mg/g)	Ratio of PBS-soluble VHH in rice seed (%)	Total accumulation of VHH in rice seed (mg/g)	Extraction efficiency of VHH in rice seed (%)
7C6	1.35	36.2	5.39	0.54	7.17	75.1
7C6-1E4	0.52	16.5	2.78	0.21	4.63	59.9

### Localization of 7C6 VHH and 7C6-1E4 VHH in Transgenic Rice

**Figure 3:**
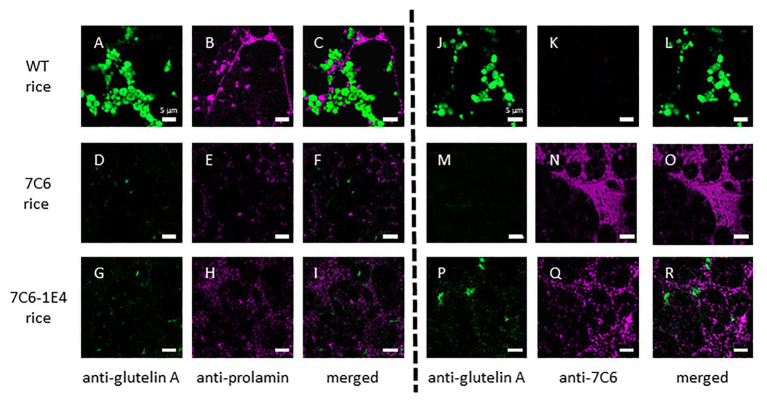
Localization of the endogenous rice proteins prolamin and glutelin and the VHHs near the sub-aleurone cell layers, as shown by double immunofluorescence staining with a combination of anti-glutelin A antibody and anti-13-kDa prolamin antibody **(A–I)** or anti-glutelin A and anti-7C6 antibody **(J–R)** in mature seeds. Panels **(A–C)** and **(J–L)** show WT seeds, **(D–F)** and **(M–O)** show MucoRice-7C6 seeds, and **(G–I)** and **(P–R)** show MucoRice-7C6-1E4 seeds. Green signal indicates the localization of glutelin, and magenta signal indicates the localization of prolamin or 7C6 VHH. Scale bars, 5 μm.

To assess the effect of RNAi on the expression of monomeric VHH 7C6 and heterodimeric VHH 7C6-1E4 in MucoRice, we investigated the distribution of VHH in rice seed endosperm tissue by immunostaining semi-thin sections with a combination of anti-glutelin antibody (mouse), anti-prolamin antibody (rabbit), and anti-7C6 antibody (rabbit; Figures 3A–R). Generally, the storage protein prolamin is localized in endoplasmic reticulum (ER)-derived granules called type I protein bodies (PBs-I); other storage proteins, such as glutelin and globulin, are in vacuole-derived granules called type II protein bodies (PBs-II). In WT rice seeds, prolamin, and glutelin were localized in different granules in the cell layer near the outer periphery of seed endosperm tissue (Figures 3A–C). In the cases of MucoRice-VHH 7C6 and 7C6-1E4, almost no prolamin and glutelin signals were observed, suggesting that RNAi successfully suppressed the expression of the endogenous rice seed proteins (Figures 3D–I; Supplementary Figure 2). Double immunostaining with anti-glutelin antibody and anti-7C6 antibody showed that monomeric MucoRice-VHH 7C6 had almost no glutelin signal (Figures 3M–O). Heterodimeric MucoRice-VHH 7C6-1E4 showed slightly weaker signals than monomeric MucoRice-VHH 7C6 because its accumulation was less than that of 7C6 (Figures 3P–R), consistent with the quantity data from SDS-PAGE (Figure 2; Table 1).

**Figure 4:**
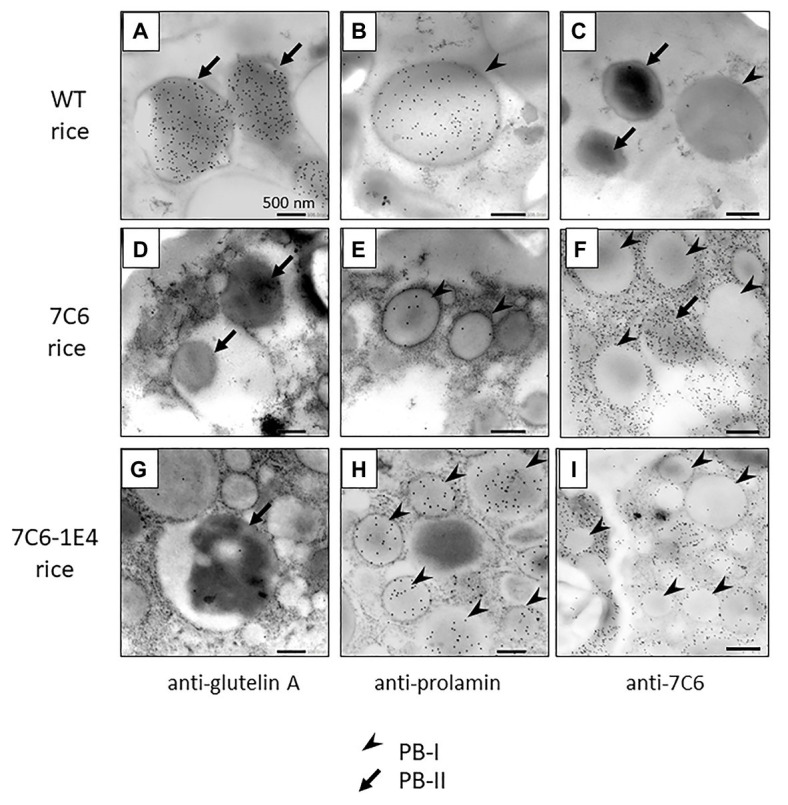
Observation of VHH distribution in premature transgenic rice seeds (14 days after flowering) of WT **(A–C)**, MucoRice-7C6 **(D–F)**, and MucoRice 7C6-1E4 **(G–I)** by immune-transmission electron microscopy. Each ultrathin section was strained with anti-glutelin A antibody **(A,D,G)**, anti-prolamin antibody **(B,E,H)**, or anti-7C6 antibody **(C,F,I)**. Arrowheads indicate type I protein bodies (PBs-I), and arrows indicate type II protein bodies (PBs-II). Scale bars, 500 nm.

To investigate the detailed localization of VHH in the endosperm tissue cells of MucoRice-VHH 7C6 and MucoRice-VHH 7C6-1E4, immuno-transmission electron microscopic analysis was performed (Figures 4A–I). In WT seeds, PBs-I formed spherical structures with a diameter of 1–3 μm, and PBs-II formed electron-dense, irregularly shaped granules (Figures 4A–C). Immunoelectron microscopic analysis of MucoRice-VHHs showed that PBs-I were smaller (less than 1 μm) than in WT, and PBs-II formation was scant or absent in both MucoRice-VHH 7C6 and 7C6-1E4 (Figures 4D–I). The VHHs were predominantly localized in the cytoplasm near starch granules and PB-IIs. These results explain the highly soluble nature of the MucoRice-VHHs in PBS (Figure 2; Table 1).

### Ability of VHHs Extracted From MucoRice (Rice Water) to Neutralize Norovirus

**Figure 5:**
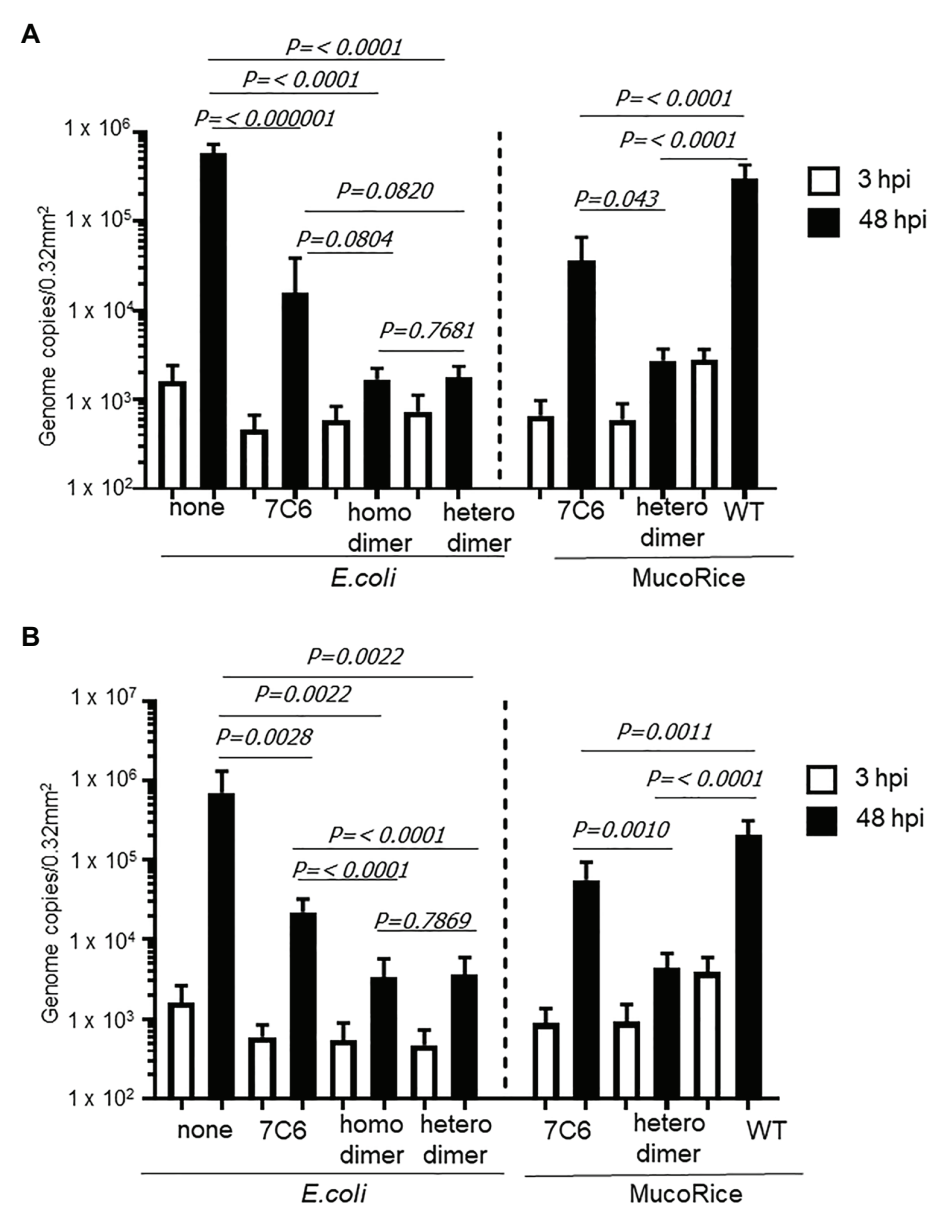
Neutralization of GII.4 Sydney **(A)** and GII.42006b **(B)** human noroviruses by VHHs from *Escherichia coli* or MucoRice. GII.4 Sydney_2012 **(A)** or GII.42006b **(B)** human norovirus genome equivalents at 2 × 10^6^ were incubated for 2 h with 5 mg of the VHH of interest before being used to inoculate human intestinal epithelial cells (IECs). Inoculation, sampling, and quantification of genome equivalents were done as described in the Materials and Methods. After inoculation and being left stand for 3 h, IECs were cultured in differentiation medium for 48 h with bile. Each value is representative of at least two independent experiments, and data are shown as the means ± 1 SD of 4–6 wells of supernatant from each culture group.

**Figure 6:**
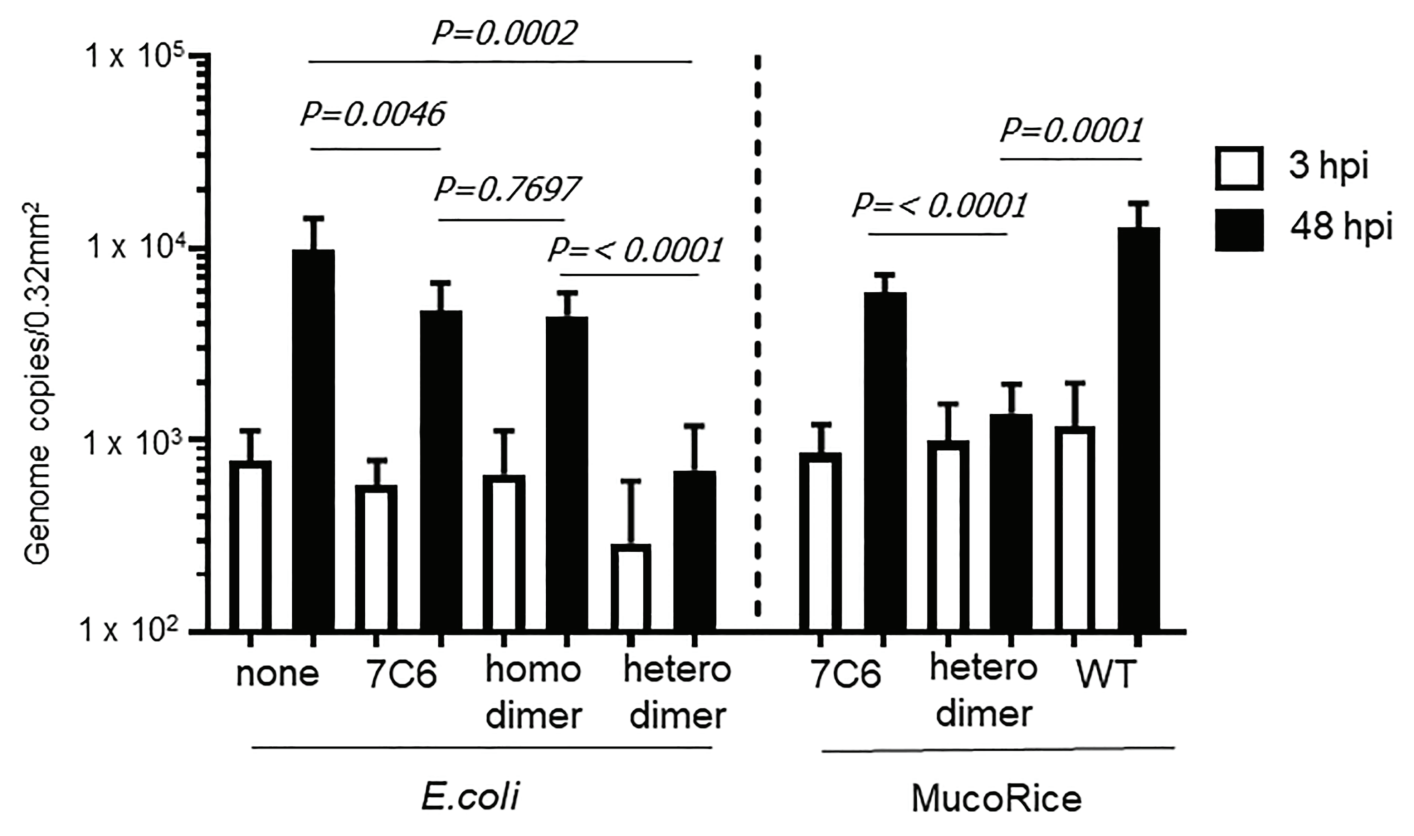
Neutralization of GII.17 human norovirus by VHHs from *E. coli* or MucoRice. GII.17 Kawasaki308 human norovirus at 2 × 10^6^ genome equivalents was incubated for 2 h with 5 mg of the VHH of interest before being used to inoculate human IECs. Inoculation, sampling, and quantification of genome equivalents were done as described in the Materials and Methods. After inoculation and being left to stand for 3 h, IECs were cultured in differentiation medium for 48 h with bile. Each value is representative of at least two independent experiments, and data are shown as the means ± 1 SD of 4–6 wells of supernatant from each culture group.

We used human IECs derived from iPSCs to test whether the monomeric (7C6) and heterodimeric (7C6-1E4) forms of VHHs extracted with PBS from MucoRice (rice water) neutralized GII.4 (Figure 5) and GII.17 (Figure 6) human noroviruses. Even though VHH 7C6 and 1E4 were produced from llamas immunized with GII.4 Sakai 08-403_2006b and GII.17 VLPs, respectively, rice water from MucoRice-VHH 7C6 and 7C6-1E4 neutralized both GII.4_2006b and GII.4 Sydney_2012 human noroviruses (Figure 5). Note that the neutralization activity of MucoRice-VHH 7C6-1E4 to both GII.4 strains was higher than that of MucoRice-VHH 7C6 (Figure 5). For comparison, we generated a recombinant VHH 7C6-7C6 homodimer and 7C6-1E4 heterodimer in *E. coli* and assessed them in the IEC norovirus neutralizing assay. Both the homomeric and heterodimeric forms of the recombinant VHHs from *E. coli* were equivalent in neutralizing activity to MucoRice-VHH 7C6-1E4 (Figure 5). Furthermore, the neutralization ability of the recombinant homodimer and heterodimeric VHHs was greater than that of recombinant VHH 7C6 monomer (Figure 5). These results show that dimerization of VHHs – regardless of whether as homodimers or heterodimers – enhanced the neutralization activity against norovirus. In addition, our data confirmed that the heterodimeric MucoRice-VHH 7C6-1E4, but not monomeric MucoRice-VHH 7C6 or the recombinant *E. coli* 7C6 homodimer, neutralized GII.17 norovirus (Figure 6). This result suggests that MucoRice-VHH 7C6-1E4 may be an attractive candidate for oral immunotherapy and prophylaxis against infections with GII.4 and GII.17 noroviruses.

### Heat Stability of VHHs Extracted From MucoRice (Rice Water)

**Figure 7:**
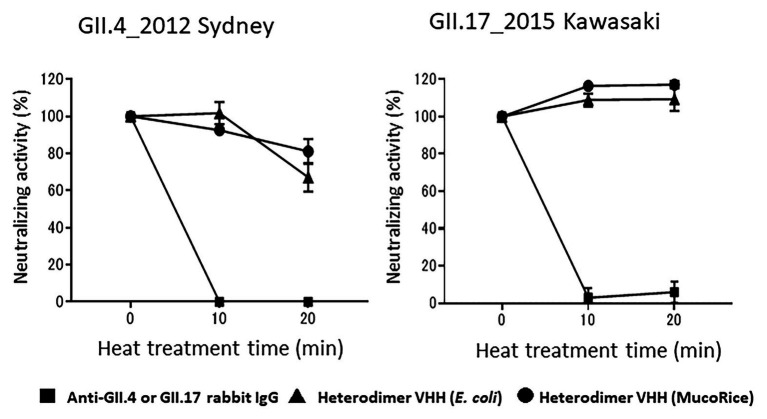
Heat stability of heterodimeric VHH. After heat treatment at 90°C for 10 and 20 min, the neutralization activity of rabbit anti-GII4_2014 virus-like particle (VLP) IgG, rabbit anti-GII.17 VLP IgG, and the heterodimeric VHHs from *E. coli* or MucoRice against GII.4_2012 Sydney and GII.17_2015 Kawasaki human noroviruses was measured in a human norovirus propagation system by using induced pluripotent stem cell (iPSC)-derived human IECs. Data are means ± 1 SD from two independent representative experiments; note that the SD is too narrow to see in the figure.

Finally, we tested whether rice water containing the MucoRice-VHH 7C6-1E4 heterodimer remained stable after heat treatment at 90°C for 20 min. Heat-treated rice water from MucoRice-VHH 7C6-1E4 retained its neutralizing activity against both GII.4 and GII.17 norovirus, whereas rabbit anti-GII.4 and GII.17 IgG antibodies lost all neutralizing activity immediately after heating (Figure 7). In particular, the neutralizing activity of rice water from MucoRice-VHH 7C6-1E4 was not at all diminished after heat treatment. These results suggest that MucoRice-VHH 7C6-1E4 heterodimer could be used during oral immunotherapy and as noroviral prophylaxis even as a rice-soup formulation.

## Discussion

An estimated 200,000 people, including 70,000 children younger than 5 years, die annually worldwide from norovirus-associated diseases (Lopman et al., 2016). East Asian outbreaks of the GII.4 Sydney and GII.17 Kawasaki strains occurred in 2012 and 2015, respectively (Maritschnik et al., 2013; Matsushima et al., 2015). Although vaccines against the GII.4 and GI.1 strains are under development currently (Kim et al., 2018; Leroux-Roels et al., 2018), other strategies involving active (e.g., vaccine) or passive (e.g., antibody/VHH) immunity to other noroviral strains (GII.17) are unavailable.

In 2018–2019, the European Medicines Agency and United States Food and Drug Administration approved Cablivi (caplacizumab; for the treatment of acquired thrombotic thrombocytopenic purpura) as the first VHH-based medicine; consequently VHHs are now attracting attention as new, safe, and effective antibody applications (Scully et al., 2019). The VHH is small (15 kDa), acid stable, and resistant to the digestive action of pepsin, making it suitable for oral antibody administration (van der Linden et al., 1999). Although a high-yield expression system for VHH (i.e., 1.0 g/L culture) has been established, we purposely adopted the rice-based system to express norovirus-specific VHHs to further advance the stable delivery of VHH in the harsh environment of the gastrointestinal tract, given that partial degradation of yeast-derived VHH occurred when it was administered *via* the oral route (Sanaei et al., 2019). According to a clinical study in Bangladesh, at least seven oral doses (50–100 mg/dose) of purified yeast-based VHH were needed to control rotaviral disease in children (Sarker et al., 2013). In this regard, MucoRice-based VHH is particularly attractive as a ready-to-use, oral antiviral product that can distributed without the need to maintain a cold chain, thus resulting in a cost-effective expression method (Tokuhara et al., 2013), given that the use of a transient expression system (Teh and Kavanagh, 2010) in *Nicotiana benthamiana* gave high expression levels but that the purification of VHH in *N. benthamiana* resulted in low yields.

In our previous study, we demonstrated that VHH 7C6 and VHH 1E4 neutralized GII.4 and GII.17 noroviruses, respectively (Yuki et al., 2020). In the current study, we adapted the rice-based expression system by using overexpression with RNAi to suppress the production of the major rice endogenous storage proteins and thus predominantly produce VHH 7C6 monomer and 7C6-1E4 heterodimer. In the case of the monomeric MucoRice-VHH 7C6, the production level (as PBS-soluble protein per gram of seed weight) was about 0.5% (Figure 2; Table 1), consistent with that of the monomeric ARP1 VHH targeting rotavirus (Tokuhara et al., 2013). Our current investigation has further demonstrated the uniqueness and advantage of the MucoRice system as an outstanding cost-effective expression system for VHHs against viruses that cause diarrheal infections (e.g., norovirus and rotavirus).

Immunohistology revealed that modifying the MucoRice-VHH expression system by adding RNAi reduced most of the endogenous prolamin and glutelin (Figure 3). Our previous study showed that PBs-I and PBs-II were distributed in the gaps between starch granules in WT rice seeds (Tokuhara et al., 2013; Abe et al., 2014; Kurokawa et al., 2014). In contrast, the diameter of the PBs-I of the RNAi-introduced rice (<1 μm) was much smaller than that in WT seeds (1–3 μm), and the structure of PBs-II had collapsed owing to RNAi suppression of prolamin and glutelin. Instead, the foreign proteins were localized in the cytosol and near the cell wall (Tokuhara et al., 2013; Abe et al., 2014; Kurokawa et al., 2014). We noted a similar phenomenon for the VHHs of 7C6-1E4 heterodimer and 7C6 monomer (Figure 4), suggesting that RNAi suppression of endogenous proteins has the potential to increase the production of the amino acids needed for synthesis of VHHs and the space available in endosperm cells, leading to high-level accumulation of VHHs in MucoRice.

We consider that the localization of the protein to the rice seed endosperm influences the solubility of VHHs, because most of the MucoRice-ARP1, which is PBS-soluble, is localized to the cytosol and PBs-II (Tokuhara et al., 2013). In our current study, the VHHs of MucoRice-7C6 monomer and MucoRice-7C6-1E4 heterodimer showed similar localizations as MucoRice-ARP1 (Figure 4), suggesting the importance of localized expression and accumulation of VHHs in the rice seed endosperm tissue for solubility in PBS. Expression of VHHs in the seeds of other plants, such as *Arabidopsis thaliana*, *N. benthamiana*, and *Nicotiana tabacum*, resulted in VHH yields of 0.01–13.1% of the total solubilized protein (Liu and Huang, 2018), whereas those of VHH 7C6 monomer and 7C6-1E4 heterodimer in MucoRice seeds were much higher, at 36.2 and 16.5%, respectively (Table 1). Furthermore, the extraction efficiency of VHH solubilized from MucoRice seeds was 75.1% for 7C6 and 59.9% for 7C6-1E4 (Table 1), resulting in the harvest of more than half of the total VHH accumulated in seeds. Our results indicate that the MucoRice system is a superior platform for expressing large quantities of VHHs.

In WT seeds, alcohol-soluble prolamin is localized in the ER lumen (Yamagata et al., 1982; Yamagata and Tanaka, 1986), and dilute acid/alkaline-soluble glutelin and salt-soluble globulin are localized in vacuoles (protein storage vacuoles; PSVs; Tanaka et al., 1980; Krishnan et al., 1986). However, owing to the influence of RNAi, the PBs-I of MucoRice were shrunken, and PBs-II were disrupted (Figure 4); VHHs consequently were localized to cytosolic regions different from those of PBs-I and PBs-II (Figures 3, 4). In a transgenic rice that expressed five tandem repeats of glucagon-like peptide 1 under the control of the GluB-1 promoter and the GluB signal peptide pSP [(GluB)-mGLP × 5 line], GLP-1 × 5 peptide was concentrated in the ER but not in PBs-I (Yamada et al., 2006). In addition, the (GluB)-mGLP × 5 line showed greater accumulation of the GLP × 5 peptide than other transgenic lines in which the GLP × 5 peptide was localized to PBs-I, PBs-II, or the intercellular compartment (Yamada et al., 2006). In the present study, the VHHs of 7C6-1E4 heterodimer and 7C6 monomer were expressed downstream of the 10-kDa signal peptide and were observed in the cytosol, in the gaps created between shrunken PBs-Is and PBs-IIs (Figure 4). We confirmed that the signal sequence of 10-kDa prolamin was cleaved correctly in MucoRice-CTB seeds; in our previous study, the same pZH2B vector as used in our current study was used to express the cholera toxin B subunit (Yuki et al., 2013). Therefore, the 10P-SP of MucoRice-VHH 7C6 and the 7C6-1E4 heterodimer likely are processed correctly in the ER. In general, ER-targeted protein is transported to protein storage vacuoles (PSV/PB-II) *via* the Golgi apparatus. However, owing to RNAi suppression of endogenous protein, VHH in the MucoRice system may accumulate in the cytoplasm between PB-I and PB-II or near the cell wall, in addition to PSV/PB-II.

The expression level of MucoRice-VHH 7C6-1E4 heterodimer was not quite half that of MucoRice-VHH 7C6 monomer (Table 1). However, in addition to its ability to neutralize GII.17 norovirus (Figure 6), MucoRice-VHH 7C6-1E4 heterodimer neutralized GII.4_2006b and GII.4 Sydney_2012 noroviruses much more strongly than did MucoRice-VHH 7C6 monomer (Figure 5). Although it was produced from llamas immunized with GII.4_2006b VLP, VHH 7C6 neutralized not only GII.4_2006b but also GII.4 Sydney_2012, a strain that mutated from GII.4_2006b and a GII.4 norovirus responsible for a recent outbreak. Although norovirus interacts with human histo-blood group antigen (HBGA) as a host attachment factor (Czakó et al., 2012), thus enabling these strains to infect humans, VHH 7C6 inhibited GII.4 Sydney_2012 norovirus most likely through direct competition for residues at the HBGA binding site or through steric hindrance in the HBGA pocket. A similar mechanism may be involved in the inhibition of GII.17 norovirus by VHH 1E4 (Yuki et al., 2020). Because the neutralizing effects of recombinant 7C6-7C6 homodimer and 7C6-1E4 heterodimer, which were produced in the *E. coli* expression system, on the GII.4_2006b and GII.4 Sydney_2012 noroviruses were nearly equal to that of the MucoRice-VHH 7C6-1E4 heterodimer (Figure 5), the dimerization of VHH may crucial for strong inhibition of norovirus. In terms of GII.17 norovirus neutralization, this result is consistent with the fact that the 7C6-1E4 heterodimer is 200 times more effective on a per-mole basis than the monomeric VHH 1E4 (Yuki et al., 2020). Covalently dimerized Anti human TNFα (hTNFα-VHH) fused with the human *κ* light-chain constant domain (anti-TNF-V_H_H_Cκ_) has been expressed in tobacco (*N. benthamiana*) leaves (Giersberg et al., 2010). This dimerization of anti-TNF-V_H_H_Cκ_ was facilitated through the formation of intermolecular disulfide bridges within the κ light-chain constant domains. Consequently, the neutralizing activity of the hTNFα-VHH dimer was 10 times higher than that of the monomeric form (Giersberg et al., 2010). These results, together with our current findings, imply that expressing VHHs as multimeric forms with a flexible linker likely will increase their avidity and synergistic effects against various viruses and other pathogens.

Variable domain of a llama heavy-chain antibody fragment monomer is heat stable at boiling (Dolk et al., 2005). We further showed here that MucoRice-heterodimeric VHH is stable at 90°C for 20 min, thus opening the possibility of not only drinking the antibody as a beverage but also consuming the antibody as a “rice soup” or porridge. Because human norovirus infection is associated with severe complications in infants, children younger than 5 years, and elderly adults, an antibody-containing rice soup or porridge may be applicable for use as a new strategy for oral immunotherapy and prophylaxis against human noroviruses.

In conclusion, we developed oral antibody-producing rice (MucoRice-VHH 7C6-1E4 heterodimer) for prophylaxis against, and treatment of, GII.4 and GII.17 noroviral infections in healthy persons of all ages and in immunocompromised patients. Because a suitable animal model for human norovirus is unavailable, we used a human norovirus propagation system in human IECs derived from iPSCs to demonstrate that the MucoRice-VHH 7C6-1E4 heterodimer neutralized GII.4 (2006b and Sydney_2012 strains) and GII.17 (Kawasaki_2015 strain) noroviruses. Rice powder and rice water containing MucoRice-VHH 7C6-1E4 offer novel approaches for the prevention and treatment of norovirus-induced diarrhea, thereby reducing medical and economic burdens in both developed and developing countries.

## Data Availability Statement

The original contributions presented in the study are included in the article/Supplementary Material; further inquiries can be directed to the corresponding author.

## Ethics Statement

All stool samples from human patients were collected under informed consent and provided by the Osaka Institute of Public Health and Nihon University School of Medicine. The study was approved by the human ethical committees of the University of Tokyo (approval number 28-40), Osaka University (approval number 28-3), the Osaka Institute of Public Health (approval number 1602-04-2), and Nihon University (approval number 29-9-1). Written informed consent for participation was not required for this study in accordance with the national legislation and the institutional requirements. The rabbit immunization experiment for the production of antibody against 7C6 was performed in accordance with the Guidelines for Use and Care of Experimental Animals and was approved by the Institutional Animal Care and Use Committee of the University of Tokyo (approval number A18-35).

## Author Contributions

AS, SK, and YY planned the experimental project. AS, SK, SS, and YY analyzed the data. AS, SK, SS, NM, YG, MU, NS, MK, HS, RN-O, HU, and KF assisted with and performed the experiments. AS, SK, SS, YY, and HK wrote the manuscript. All authors contributed to the article and approved the submitted version.

### Conflict of Interest

The authors declare that the research was conducted in the absence of any commercial or financial relationships that could be construed as a potential conflict of interest.

## Footnotes

^1^https://web.expasy.org/cgi-bin/protparam/protparam

^2^https://imagej.net/Fiji
