# Supplementary material for: Development of Antibody-Fragment–Producing Rice for Neutralization of Human Norovirus
Source: Front Plant Sci. 2021 Feb 23;12:639953. doi: 10.3389/fpls.2021.639953 (PMC8047661; doi:10.3389/fpls.2021.639953)
Supplement: Supplementary file 1 [file Data_Sheet_1.pdf]

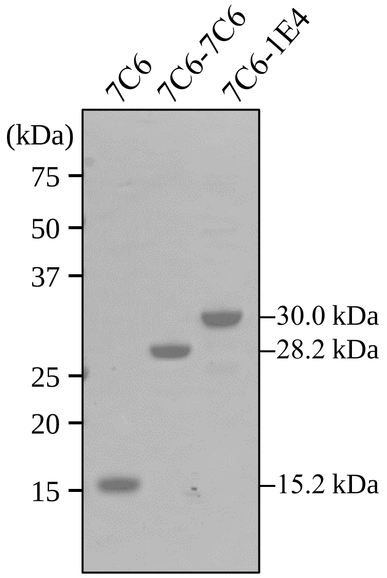

Supplementary Figure 1. Image of SDS–PAGE gel containing recombinant VHH protein expressed by *Escherichia coli*. Each lane contains 1 µg of purified protein.

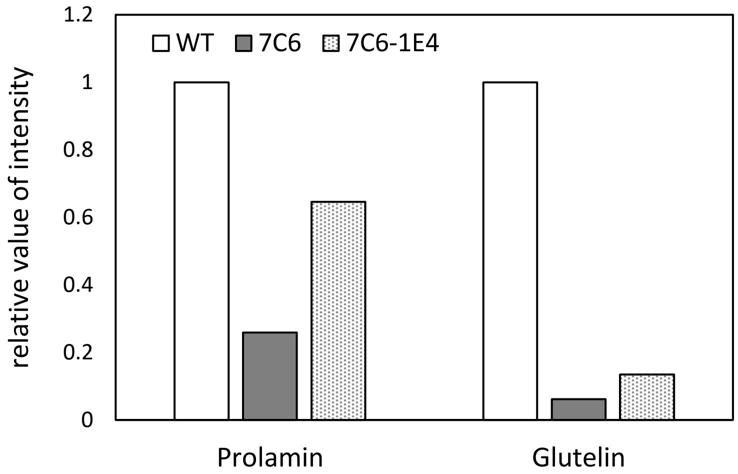

Supplementary Figure 2. Relative fluorescence intensity values of 13-kDa prolamin and glutelin when the average WT intensity was defined as 1. As described in the Materials and Methods section, the images in **Figure 3A**, **B**, **D**, **E**, **G,** and **H** were used for this measurement.

Supplementary Table 1. VHHs raised by using human norovirus VLPs

VHH construct	VLP used	Neutralization to HuNoV
7C6	GII.4 Sakai08-403_2006b	GII.4 2006b, GII.4 Sydney_2012
1E4	GII.17 Kawasaki 308 (OsakaF B16421, 2017)	GII.17(Kawasaki 308)

Supplementary Table 2. Live human noroviruses used

Strain designation	Strain	Titer (genome equivalents/μL)
GII.4_2006b	Hu/GII.4_2006b/osaka13499/2015/JP	1.7×10^6
GII.4 Sydney_2012	Hu/GII.Pe GII.4/OsakaFB1753/2017/JP	2.2×10^7
GII.17 Kawasaki 308	Hu/GII.P17/OsakaFB16421/2017/JP	5.8×10^7
